# Determining the Optimal N Input to Improve Grain Yield and Quality in Winter Wheat With Reduced Apparent N Loss in the North China Plain

**DOI:** 10.3389/fpls.2019.00181

**Published:** 2019-02-22

**Authors:** Geng Ma, Weixing Liu, Shasha Li, Panpan Zhang, Chenyang Wang, Hongfang Lu, Lifang Wang, Yingxin Xie, Dongyun Ma, Guozhang Kang

**Affiliations:** ^1^College of Agronomy, Henan Agricultural University, Zhengzhou, China; ^2^State Key Laboratory of Wheat and Maize Crop Science, Henan Agricultural University, Zhengzhou, China; ^3^National Engineering Research Centre for Wheat, Henan Agricultural University, Zhengzhou, China

**Keywords:** wheat, grain yield, protein content, N application rate, N use efficiency, apparent N loss

## Abstract

Excessive or improper nitrogen (N) application rates negatively affect crop production and thereby environmental quality, particularly for winter wheat production in the North China Plain. Therefore, it is very important to optimize N fertilizer input to balance grain yield, environmental risk, and benefits under irrigated conditions. Three long-term stationary field experiments including five N levels, from 0 to 300 kg ha^-1^ [0 (N0), 90 (N90), 180 (N180), 240 (N240), and 300 (N300) kg ha^-1^] were carried out to investigate the effects of N regime on wheat yield, photosynthesis, and N balance at different sites. The grain yield and protein content increased quadratically with N rate, and the maximum values were 8087 kg ha^-1^ and 13.9% at N application rates of 250 and 337 kg N ha^-1^, respectively. N application increased the photosynthetic fluorescence parameters (Pn, Gs, and Tr) and N metabolism enzyme activities (NR and GS) which then increased grain yield. The leaching of soil nitrate into the deeper soil layers ( > 100 cm) increased with higher N fertilization and experimental years. The partial factor productivity (PFPN) was decreased by N because the apparent N loss increased with N application rate. In order to balance grain yield, N use efficiency (NUE), and N loss, the recommended N rate should be 120–171 kg N ha^-1^, and the corresponding yields and apparent N loss were 7278–7787 ka ha^-1^ and 22–37 kg ha^-1^, respectively.

## Introduction

As the largest wheat producer and consumer in the world, China produced about 115 million tons of wheat on a planting area of 24.3 million hectares (ha) in 2010 (FAO, 2013). The Huang-Huai-Hai Plain is one of the most important food production regions in China, where more than 60% of the wheat is planted and the irrigated area accounts for approximately 70% of the total cultivated area (NBS, 2013). High yields will be necessary and important to feed the growing population on the limited cropland in the coming decades (Zhang et al., 2013). Also, grain protein content is one of the most important quality traits of wheat, especially in countries or regions in which the dietary protein intake depends largely on the amount of protein in cereal grains.

Generally, nitrogen (N) is considered to be the single most important factor for determining wheat productivity and grain quality in the current cropping systems employed around the world (Triboi et al., 2000; Sinclair and Rufty, 2012). Therefore, to ensure the necessary high yields, protein content, and good economic benefit, farmers tend to apply excessive N fertilizer due to a lack of knowledge about how to use N effectively and how to prevent groundwater pollution from NO_3_–N. In fact, excessive N application does not significantly improve crop yields, and it can decrease N use efficiency (NUE) and also cause serious environmental problems due to the loss of a large amount of applied N (∼180 kg ha^-1^ yr^-1^) into the environment (Vitousek et al., 2009). The extent of soil nitrate accumulation (SNA) is so high that 55% of farmers find that it is not necessary to apply N fertilizers before sowing (Cui et al., 2010), as revealed from data on wheat yield response. Therefore, nitrate accumulation in the soil has been increasing attention.

At present, crop N use efficiency (NUE) is low because the input rate of chemical N fertilizer greatly surpasses crop needs (Ju et al., 2009). It has been previously reported that crop yields are correlated with NO_3_-N levels in the soil at harvest (Miao et al., 2014). Some studies have examined the relationship between wheat grain yield and N application rate at specific locations or at different scales (Valkama et al., 2013; Gaudin et al., 2015) using several models, and the quadratic model has been most commonly used in China (Hartmann et al., 2015). Recently, several studies have been carried out on nitrate accumulation at harvest in this intensive crop production region (Delin and Stenberg, 2014; Wang et al., 2015). However, these were all short-term studies that failed to identify the variations and characteristics of yield and nitrate accumulation and distribution in the soil profile under the specific climatic conditions and the cropping system used in this area.

Therefore, we initiated a field experiment in 2011 with winter wheat grown at different N rates on the same plots each year. The objectives of the investigation were to: (1) study the long-term effects of varying levels of N fertilization on wheat yield, protein content, and photosynthetic characteristics, (2) ascertain residual nitrate accumulation that occurs in the different soil layers and quantify apparent N loss for the different N applications, and (3) determine an optimal N application rate to balance yield, benefit, NUE, and environmental cost on the North China Plain.

## Materials and Methods

### Experimental Site

The field experiments were conducted at three locations in Henan Province, which is a typical Huang-huai crop planting area in China for winter wheat production. From 2011–2016, experiments were simultaneously carried out at Wenxian (34°92′ N, 112°99′ E) and Zhengzhou (34°47′N,113°38′E), where the annual mean temperatures are 13.0° and 14.3°C and the annual precipitation levels are 650 mm and 605 mm, respectively. For the 2013–2017 growing seasons, the experiments were performed at Kaifeng Experimental Farm (34°41′N,114°49′E), where the annual mean temperature and precipitation level are 14.5°C and 627 mm, respectively. Supplementary Figure S1 shows the precipitation distribution during the wheat growing seasons at the three experimental sites. The physical and chemical properties of the 0–20 cm soil layers at the experimental sites are given in Supplementary Table S1. The natural conditions and agricultural production levels at the three experimental sites are typical for the Huang-Huai wheat production area and represent standard high-yield agricultural production conditions for winter wheat.

### Experimental Design and Fertilizer Treatments

‘Yumai 49–198’, a high-yielding wheat cultivar that is widely planted in the Huang-Huai wheat production area, was used in this study. The field experiments were designed with four different N fertilizer rates (0, 180, 240, and 300 kg ha^-1^; designated N0, N180, N240, and N300, respectively) in Wenxian and Kaifeng, while the experiment had five N rates (0, 90, 180, 240, and 300 kg ha^-1^) in Zhengzhou. Individual plots were 6.1 m × 2.5 m (15.25 m^2^) in Wenxian and 7 m × 2.9 m (20.3 m^2^) in Zhengzhou and Kaifeng, with three replicates in a randomized design. All plots were irrigated at the jointing and booting stages with 75 mm each time; the amount of water used was calculated using a water meter. Nitrogen fertilizer was applied as urea (46%), and phosphorus (P) and potassium (K) fertilizer as calcium superphosphate (15%) and potassium chloride fertilizer (60%) at rates of 150 (P_2_O_5_) and 120 (K_2_O) kg ha^-1^, respectively. Half the amount of nitrogen, and all of the phosphorus and potassium fertilizer, was spread by hand before plowing at the time of sowing. Additional N fertilizer was applied at the jointing stage in selected plots. The management practices for controlling pests, diseases, and weeds complied with local practices for high-yield wheat production.

### Sampling and Sample Analysis

At maturation stage, wheat was hand-harvested from a 6 m^2^ area (3 m × 2 m) in the middle of each plot. The grain was weighed after air-drying, and the grain weights at corresponding moisture contents were recorded and expressed against a standard moisture content of 13%. Grain protein was calculated by multiplying the nitrogen content by 5.7, and nitrogen content was measured using a nitrogen analyzer (Kjeltec 2300, FOSS, Sweden) according to the ICC Standard Method 105/2. Soil samples were collected from 0–20, 20–40, 40–60, 60–80, and 80–100 cm soil depths for determination of NO_3_^-^-N before sowing and after harvest. Soil samples (5 g) were extracted in 50 ml 1M KCl and shaken for 1 h (Li et al., 2012). After filtration, the extracts were immediately measured for nitrate-N concentration using a high-resolution digital colorimeter AutoAnalyzer3 (AA3; SEAL Company, Germany).

Chlorophyll was estimated in crude flag leaf extracts at anthesis using the method of Arnon (1949). Briefly, acetone (80%) was used for extraction of chlorophyll and particulate impurities were removed by centrifugation. Chlorophylls a and b were calculated by measuring absorbance at 663 and 645 nm, respectively, and the total chlorophyll content is the sum of chlorophylls a and b.

Photosynthetic parameters and chlorophyll fluorescence were measured using an LI-6400 Portable Photosynthesis System (LI-COR, Lincoln, Nebraska, United States) and a portable chlorophyll fluorometer (FMS 2.02; Hansatech, United Kingdom) under field conditions at anthesis stage in the growing season of 2015–2016. Intact leaves of fresh wheat plants from each treatment were selected to measure the following photosynthetic gas exchange parameters: photosynthetic rate (Pn), stomatal conductance (Gs), internal CO_2_ concentration (Ci) and transpiration rate (Tr). The measurements were taken from 9:00 am to 11:00 am to avoid the midday decrease in photosynthesis. was measured using Minimal fluorescence, F_0_, was measured in 30 min dark-adapted leaves and maximal fluorescence, F_m_, was measured in the same leaves under full light-adapted conditions. Maximal variable fluorescence (F_v_ = F_m_-F_0_) and the photochemical efficiency of PSII (F_v_/F_m_) for dark-adapted leaves were also calculated from the measured parameters (Maxwell and Johnson, 2000). In light-adapted leaves (for 15 min), the steady state fluorescence yield (F_s_′), maximal fluorescence (F_m_′) after a 0.8 s saturating white light pulse, and minimal fluorescence (F_0_′) were measured when the actinic light was turned off, and further calculations were made by using the equation F_0_′ = F_0_/(F_v_/F_m_+F_0_/F_m_′) (Oxborough and Baker, 1997). Values for quenching due to non-photochemical dissipation of absorbed light energy (qN) was determined at each saturating pulse using the equation qN = (F_m_-F_m_′)/F_m_′. The coefficient for photochemical quenching, qP, which represents the fraction of open PSII reaction centers, was calculated as qP = (F_m_′-F_s_′)/(F_m_′-F_0_′) (Maxwell and Johnson, 2000), The photochemical efficiency of photosystem II (ΦPSII) was calculated as follows:

(1) $$\begin{array}{c} \Phi PSII=(F_{m}^{\prime }-F_{s}^{\prime })/F_{m}^{\prime } \end{array}$$

Enzymatic activity in the flag leaves was analyzed at 7-day intervals from 0 to 28 days after anthesis (DAA). Enzymes were extracted from leaf material stored at -80°C. All extractions were performed at 4°C. Mg^2+^-dependent nitrate reductase (NR) activity was measured according to the protocol described by Ferrario-Mery et al. (1998). Glutamine synthetase (GS) activity was measured using the synthetase assay based on the method described by Lea et al. (1990).

The soil nitrate accumulation (SNA; kg N ha^-1^) in each soil layer was calculated as follows:

(2) $$\begin{array}{c} SNA=C_{N}\times BD\times SD\times 0.1 \end{array}$$

Where *SD* is the soil layer thickness (cm), BD is the soil bulk density (g cm^-3^), C_N_ is the soil NO_3_-N concentration (mg kg^-1^) of the corresponding layer, and 0.1 is the conversion coefficient.

The apparent N mineralization rate (N_min_) during the wheat growing season was calculated as the difference between N output (plant N uptake plus residual soil N) and the N input (initial soil N in the 0–100cm soil layers) in the no-N (N0) plots (Meisinger, 1984):

(3) $$\begin{array}{c} N_{\min}=soil\;N_{\mathrm{end}}+N_{\mathrm{uptake}}-soil\;N_{initial} \end{array}$$

Apparent N losses during the wheat growing season were calculated as the difference between N input (N fertilizer application rate plus initial soil N plus apparent N mineralization) and N output (plant N uptake plus residual soil N) in plots where N was applied (Ma et al., 1999).

(4) $$\begin{array}{c} N_{\mathrm{loss}}=N_{\mathrm{fer}}+soil\;N_{\mathrm{initial}}+N_{\min}-plant\;N_{\mathrm{uptake}}-soil\;N_{\mathrm{end}} \end{array}$$

Here, soil N_initial_ and N_end_ are the SNA within the top 100 cm of the soil profile before planting and after harvest, respectively; plant N_uptake_ represents N accumulation in the above-ground biomass at harvest; and N_fer_ is the N application rate in kg ha^-1^.

Two indicators were used to evaluate NUE: (1) the partial factor productivity of N (PFPN), and (2) the agronomic efficiency of N (NAE) were calculated with the following formulas:

(5) $$\begin{array}{c} PFPN(kgkg^{-}{}^{1})=\frac{Y_{N}}{A_{N}} \end{array}$$

(6) $$\begin{array}{c} NAE(kgkg^{-}{}^{1})=\frac{Y_{N}-Y_{0}}{A_{N}} \end{array}$$

Where Y_N_ (kg ha^-1^) is the grain yield from treatments with N fertilizer, Y_0_ (kg ha^-1^) is the grain yield from the treatment without N fertilizer, and A_N_ (kg ha^-1^) is the amount of N fertilizer applied (Yang et al., 2017).

The relative economic return was calculated as: (wheat price × wheat yield increment)-(N fertilizer price × increment in amount of N fertilizer). The average prices of wheat and N fertilizer were 0.340 and 0.623 USD kg^-1^ (1 US dollar = 6.2 Chinese Yuan Renminbi) over the past two decades, computed from China’s food information network^1^ and China’s fertilizer network^2^, respectively.

### Statistical Analysis

The data were analyzed using two-way analysis of variance (ANOVA) as implemented in the SPSS software package (Version 17.0) to test whether significant differences existed between the treatments and years. The means for treatments and years were compared with the least significant difference (LSD) test at the 0.05 probability level (at p ≤ 0.05). The relationships between yield, cumulative NO_3_-N, and N application rate were analyzed using regression or stepwise regression analyses to determine the best-fit equations. The graphs were prepared using Sigmaplot 12.3.

## Results

### Grain Yield and Protein Content

**FIGURE 1:**
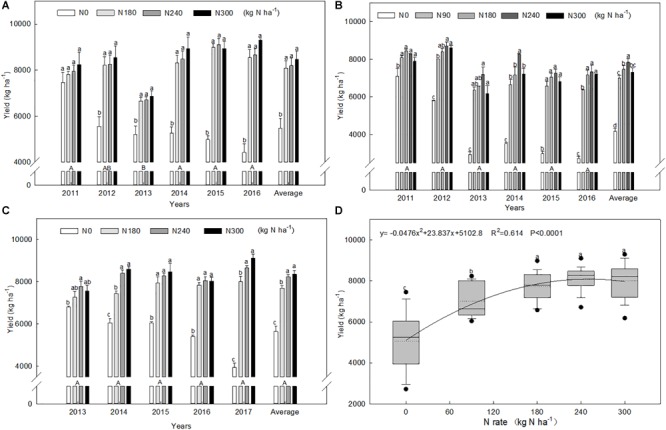
Grain yields of winter wheat at different N application rates for each experimental year in Wenxian **(A)**, Zhengzhou **(B)**, and Kaifeng **(C)**, and their correlation with N application rate **(D)**. Bars **(A–C)** denote the standard errors. Different uppercase letters indicate significant differences among years, and lowercase letters denote significant differences among N rates at *p* < 0.05 using the LSD test in SPSS. Solid and dashed lines, box boundaries, and bars and dots inside or outside of the boxes **(D)** represent the median and mean values, the 25th and 75th, the 10th and 90th, and < 5th and > 95th percentiles of all data, respectively. These symbols are the same used in Figure 2.

We found that grain yield in wheat is significantly affected by N application rate. Compared with the N0 treatment, the N180, N240, and N300 treatments increased grain yield by 47.6%, 49.6%, and 54.5% in Wenxian (Figure 1A) and 36.2%, 45.8%, and 47.9% in Kaifeng (Figure 1C), respectively. The N90, N180, N240, and N300 treatments increased yield by 67.7%, 78.7%, 87.6%, and 75.1%, respectively, compared with the N0 treatment in Zhengzhou (Figure 1B). In the first growing season, wheat grain yield in the N0 treatments were 4.5%, 15.8%, and 6.3% less than in the N180 treatments in Wenxian, Zhengzhou, and Kaifeng, respectively. However, the N0 wheat yields in the last experimental year were significantly less by 48.3%, 62.1%, and 50.8% compared to the N180 treatments in Wenxian, Zhengzhou, and Kaifeng, respectively. The average yields of N rates of 0, 90, 180, 240, and 300 kg ha^-1^ were 5102, 7010, 7751, 8089, and 8047 kg ha^-1^ respectively, across all the different experimental seasons and sites (Figure 1D). In all years and locations, regression analysis showed that wheat grain yield increased quadratically with N rate, with a maximum value of 8087.1 kg ha^-1^ that corresponded to a N application rate of 250.4 kg ha^-1^ under these experimental conditions.

**FIGURE 2:**
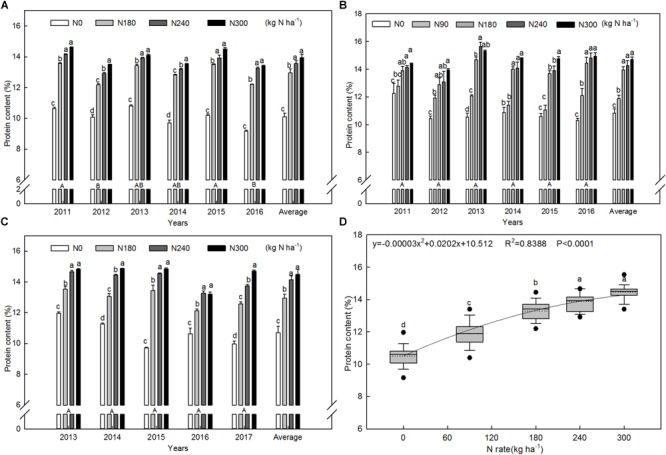
Grain protein content of winter wheat grown at different N application rates for all experimental years at Wenxian **(A)**, Zhengzhou **(B)**, and Kaifeng **(C)** and their correlation with N application rate **(D)**.

Similarly, the grain protein content increased with increases in the N rate in most years (Figure 2). The average of the N treatments increased by 29.6% compared with the N0 control. The average protein contents were 10.54%, 11.89%, 13.29%, 13.98%, and 14.37%, respectively, at N rates of 0, 90, 180, 240, and 300 kg ha^-1^ across all growing seasons and locations. Regression analysis (Figure 2D) showed that protein content increased quadratically with N rate, with a maximum value of 13.9%, corresponding to a N application rate of 336.7 kg ha^-1^ under these experimental conditions.

### Leaf Photosynthesis and Chlorophyll Fluorescence Parameters

The photosynthetic and fluorescence characteristics of flag leaves at anthesis in 2015–2016 growing season are shown in Table 1. The highest chlorophyll content was observed when the wheat was supplied with 240 kg N ha^-1^, and the N0 treatments had the lowest chlorophyll contents at all sites. There was no significant difference in the chlorophyll content of flag leaves in the N240 and N300 treatments. The Pn, Gs, and Tr of flag leaves at anthesis in the N0 treatment were significantly lower than in the other N treatments, and the Ci was highest in the N0 treatment at all three sites. Chl fluorescence was affected by N application rate, especially ΦPSII and qN under these experimental conditions. The N180 treatment had the highest ΦPSII, which was significantly higher than in the N0 treatments at all sites. However, there were no significant differences in the F_v_/F_m_ and qP values among the four treatments in Wenxian. For the N240 treatments, the Zhengzhou and Kaifeng sites had the largest qP and F_v_/F_m_, respectively, and they were significantly larger than in the N0 treatments.

**Table 1:** Chlorophyll content, photosynthesis (Pn, Gs, Ci, and Tr), and chlorophyll fluorescence (Φ_PSII_, F_v_/F_m_, qP and qN) parameters in flag leaves of wheat plants grown under different N application rates at anthesis in Wenxian, Zhengzhou, and Kaifeng (2015–2016).

Site	N rate (kg ha^-1^)	Chlorophyll content (mg g^-1^ FW)	Pn (μmol CO_2_ m^-2^ s^-1^)	Gs (mol H_2_O m^-2^ s^-1^)	Ci (μmol CO_2_ mol^-1^)	Tr (mmol H_2_O m^-2^ s^-1^)	Φ_PSII_	F_v_/F_m_	qP	qN
Wenxian	0	2.15c	16.81b	0.57b	244.33b	6.71b	0.83bc	0.86a	0.99a	0.15c
	180	2.69b	25.26a	0.61b	195.64b	7.15ab	0.87a	0.88a	0.99a	0.17bc
	240	3.07a	25.93a	0.76ab	182.52b	7.55ab	0.84bc	0.87a	1.00a	0.22b
	300	2.91ab	26.98a	0.86a	169.63bc	8.14a	0.82c	0.86a	1.00a	0.25a
Zhengzhou	0	1.75c	15.68c	0.32c	314.33a	6.52b	0.81b	0.86a	0.91b	0.11b
	90	2.35b	18.15b	0.43b	310.12a	6.69ab	0.82ab	0.86a	0.92b	0.11b
	180	2.75ab	20.36ab	0.47b	296.45ab	6.97a	0.84a	0.86a	0.96a	0.13ab
	240	2.98a	23.95a	0.66a	274.43b	7.15a	0.81b	0.88a	0.99a	0.16a
	300	2.62ab	21.55ab	0.51ab	289.60ab	7.22a	0.83ab	0.88a	0.96a	0.15a
Kaifeng	0	1.98c	16.52b	0.36c	259.67a	6.65b	0.79b	0.86c	0.93a	0.12b
	180	2.63b	22.63a	0.46b	235.28b	6.79b	0.83a	0.88bc	0.97a	0.22a
	240	3.13a	23.26a	0.58a	231.47b	7.43ab	0.82a	0.90a	0.95a	0.16ab
	300	2.86ab	21.85a	0.46b	243.23ab	7.73a	0.83a	0.89ab	0.94a	0.13b

*The lowercase letters following the values within a column indicate significant differences among N treatments at *P* < 0.05 based on the LSD test.*

### NR and GS Activities in Flag Leaves

**FIGURE 3:**
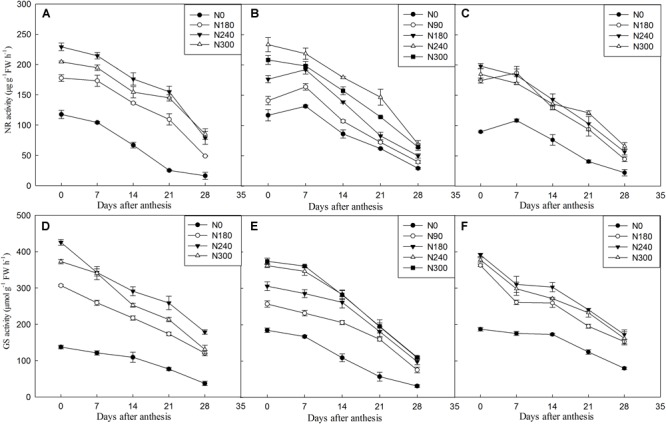
Nitrate reductase (NR) and glutamine synthetase (GS) activities in flag leaves in winter during grain filing under different N application rates at Wenxian **(A,D)**, Zhengzhou **(B,E)**, and Kaifeng **(C,F)** in 2016. Horizontal bars represent the standard errors of the mean.

Nitrate reductase (NR) is the key enzyme of N assimilation in plants, and glutamine synthetase (GS) is a multifunctional enzyme at the center of N metabolism that plays an important role in the regulation of N metabolism. Changes in the physiological markers of the flag leaf after anthesis are shown in Figure 3. This study was performed on five different sampling dates following anthesis. The results showed that the NR and GS activities of the flag leaves in the N0 treatment were significantly lower than in the other treatments from 0 to 28 DAA at all three sites. The NR activities of flag leaves in the N240 treatments were higher than for flag leaves in the other N treatments (N90, N180, N300) from 0 to 21 DAA in Wenxian (Figure 3A) and Zhengzhou (Figure 3B). The changes in flag leaf GS activity were similar to those found for NR activity.

### Soil Nitrate Accumulation (SNA)

**FIGURE 4:**
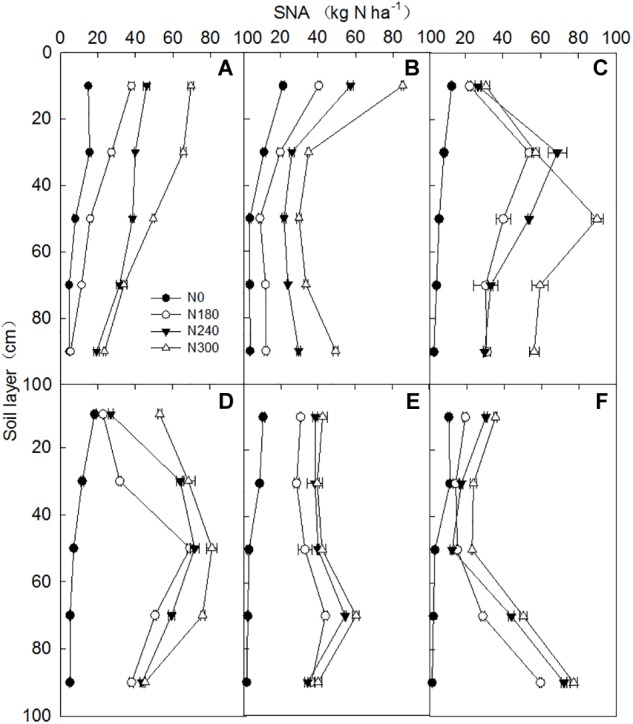
Nitrate distribution in the 0–100 cm soil layers after the winter wheat harvest for plants grown under different N application rates during the harvest years 2011–2016 in Wenxian. Panels **(A), (B), (C), (D), (E)**, and **(F)** show the nitrate distribution for the different N rates in 2011, 2012, 2013, 2014, 2015, and 2016, respectively. Horizontal bars represent the standard errors of the mean.

The results for SNA in the three experimental sites were similar, so only the soil profile distribution of SNA for the six wheat cropping seasons in Wenxian is shown in Figure 4. SNA increased significantly with increasing N application rate and was also observed in the N0 treatment. The SNA in each soil layer increased with N rate, and the highest SNA of all the treatments occurred in the surface soil layer (0-20 cm) in 2011 and 2012 (Figure 4A,B). The SNA decreased in the 20–40 cm soil layer but increased in the 40–60 cm soil layer and peaked during 2013 and 2014 (Figure 4C,D). Also, the SNA peak was at a lower depth (80–100 cm) in last two cropping years (Figure 4E,F), and the total SNA in the 0–100 cm soil layer was less than it was before the growing season in 2015 and 2016. The yearly SNA in the 0–100 cm soil layer gradually increased until the fourth experimental year (2014), and was 61.9, 175.3, 220.3, and 268.8 kg ha^-1^, respectively, for the 0, 180, 240, and 300 kg N ha^-1^ application rates. The SNA gradually decreased in the last two years (2015 and 2016) by 71.3, 25.2, 20.0, and 16.4% respectively, compared with 2014 at the 0, 180, 240, and 300 kg N ha^-1^ application rates.

### N Use Efficiency (NUE)

PFPN and NAE for winter wheat varied among treatments and growing seasons at the Wenxian site (Table 2). Both N and experimental year, and their interaction, had significant effects on PFPN and NAE. The higher the nitrogen fertilizer application, the lower the value of PFPN obtained each year. Compared with the N180 treatment, the PFPN of the N240 and N300 treatments decreased significantly by 24.5 and 37.4%, respectively. In all years except for 2013, the average PFPN values in the different treatments across all N levels increased gradually with advancing experimental years; the PFPN in 2016 increased by 10.3% compared with that in 2011. Similarly, the NAE for the N240 and N300 treatments decreased significantly by 23.5% and 31.9% on average compared with the N180 treatment, respectively. The NAE in 2016 increased 7.5-fold compared with that in 2011.

**Table 2:** Partial factor productivity of N (PFPN) and agronomic efficiency of N (NAE) for winter wheat grown at different N application rates in Wenxian for harvest years 2011-2016.

Year	PFPN (kg kg^-1^)			Average	NAE (kg kg^-1^)			Average
	N180	N240	N300		N180	N240	N300	
2011	43.36d	33.07c	27.44b	34.62f	1.94d	2.01d	2.59c	2.18d
2012	45.69c	34.42bc	28.47ab	36.19c	16.79b	12.75b	11.13b	13.56b
2013	36.94e	27.99d	22.86c	29.26e	6.07c	4.84c	4.34c	5.08c
2014	47.10b	34.61bc	29.78ab	37.16bc	17.93b	12.74b	12.28b	14.31b
2015	49.93a	37.96a	29.78ab	39.22a	22.26a	17.21a	13.17ab	17.55a
2016	47.49b	36.11ab	30.99a	38.20ab	22.46a	17.34a	15.98a	18.59a
Average	45.08A	34.03B	28.22C		14.57A	11.15B	9.92C	
**Analysis of variance(F)**								
N Rate	1091.83^∗∗∗^				41.35^∗∗∗^			
Year	94.61^∗∗∗^				160.53^∗∗∗^			
N Rate × Year	2.94^∗∗^				3.95^∗∗∗^			

*The lowercase letters following the values within a column indicate significant differences among years, and the uppercase letters within a row indicate significant differences among N treatments at *P* < 0.05 basedon to the LSD test. ^∗∗∗^*P* < 0.001; ^∗∗^*P* < 0.01; ^∗^*P* < 0.05; ns: *P* > 0.05. The symbols used top show significance are used in the other tables.*

### Apparent N Balance

Table 3 shows that the initial N accumulation before sowing, the wheat plant N uptake, and the SNA all increased with increasing N rate. Soil N mineralization during the wheat growing period varied across the sites, and was 75.56, 58.58, and 94.63 kg ha^-1^ in Wenxian, Zhengzhou, and Kaifeng, respectively. With the addition of N to the soil before sowing, the amount of N supplied by the soil can reach 227-365 kg ha^-1^, which is only slightly below the total nitrogen requirement of wheat during the growth stage. These results showed that it is feasible to reduce the amount of applied N in these three different sites. The averages for the three sites were 14.66, 43.38, 61.58, and 96.14 kg ha^-1^ for the N90, N180, N240, and N300 treatments, respectively. These results showed that the low N application rate would lead to a decrease in soil fertility under high-yield conditions, while the proper N application would be beneficial to the balance of inorganic N in the soil.

**Table 3:** Average initial N content before sowing( soil N_initial_), N uptake by the plant(plant N_uptake_), residual N after harvest (soil N_end_), N mineralization(N_min_), N loss(N_loss_), and N loss rate for all study years at the three planting sites.

Site	N rate (kg ha^-1^)	Soil N_initial_ (kg ha^-1^)	Plant N_uptake_ (kg ha^-1^)	Soil N_end_ (kg ha^-1^)	N_min_ (kg ha^-1^)	N_loss_ (kg ha^-1^)	N loss rate (%)
Wenxian	0	64.28c	105.73c	38.11d	79.56	0	—
	180	213.62b	272.75b	148.78c	79.56	51.65ab	28.69b
	240	239.47b	289.76b	199.27b	79.56	70.01b	29.17ab
	300	285.82a	315.41a	254.69a	79.56	95.28a	31.76a
Zhengzhou	0	91.94c	112.38c	38.14d	58.58	0	—
	90	128.51bc	205.54b	56.89cd	58.58	14.66c	16.29c
	180	168.56b	260.28a	107.91c	58.58	38.95bc	21.64b
	240	186.99ab	277.46a	148.75b	58.58	59.36b	24.73b
	300	216.93a	271.62a	214.76a	58.58	89.13a	29.71a
Kaifeng	0	109.21c	155.02c	48.77d	94.63	0	—
	180	133.63b	253.01b	115.71c	94.63	39.54b	21.97b
	240	185.36a	295.69a	168.94b	94.63	55.36b	23.07b
	300	211.87a	308.06a	194.42a	94.63	104.02a	34.67a

*The lowercase letters following the values within a column indicate significant differences among N treatments at *P* < 0.05 based on the LSD test.*

## Discussion

N fertilizer is a crucial factor in wheat production; it results in a significant increase in grain yield and grain protein content compared to no-N treatment (Ercoli et al., 2013), and both yield and protein content are increased to a comparable extent by N application (Zhang et al., 2016). In our study, the response of grain yield and protein content to N rates fitted a quadratic model, and N rate can explain 61% of the variation in yield and 84% of the variation in protein content (Figure 1D, 2D). Based on the quadratic model, maximum grain yield and protein content were obtained with 250 and 337 kg N ha^-1^, respectively. Similar to the findings of Abad et al. (2004), we found that grain yield reached a plateau with lower N application rates than did protein content. Thus, to a certain extent, N application is an efficient way to increase grain protein content without causing yield reductions.

Enhanced grain yields have been consistently reported to be associated with changes in photosynthetic characteristics, such as Pn, Gs, Ci, and Tr (Dahal et al., 2014). Under well-watered conditions, the Pn and Gs of plants grown under high-N nutrition conditions were greater than those of plants grown under lower-N nutrition (Shangguan et al., 2000). Compared with the N-unfertilized treatment (0 kg N ha^-1^), the Gs in the fertilized treatments was higher by an average of 27% (Dordas and Sioulas, 2008). We found that the Pn, Gs, and Tr were increased significantly by N application. Also, the Pn and Gs at the different sites were ranked in the order Wenxian > Kaifeng > Zhengzhou, and this trend was consistent with the average yield. These results indicate that N application rate affects yield formation by regulating photosynthetic parameters. In sunflower, N deficiency did not significantly change F_v_/F_m_, indicating that there was no reduction in PS II efficiency (Ciompi et al., 1996). Our results also showed that there was no significant change in F_v_/F_m_ in Wenxian and Zhengzhou in all treatments. However, the decrease in F_v_/F_m_ is remarkable in Kaifeng, and it might inhibit photochemical activities and potential photosynthetic activity in PSII. In our study, we observed that there was a significant reduction of ΦPSII in the N0 treatment, indicating a decline in the electron transport activities of PSII. These data suggest that N deficiency can lead to a reduction in photochemical efficiency in wheat leaves at anthesis. Furthermore, an excess supply of N is not conducive to the effective utilization of trapped light energy.

NR has long been considered to be the rate-limiting step in nitrate assimilation (Masclaux-Daubresse et al., 2010). The GS enzyme is responsible for the first step of ammonium assimilation and transformation into glutamine, and it exists in both cytosolic (GS1) and chloroplastic (GS2) forms that are encoded by 3-5 genes and one gene, respectively (Lea and Miflin, 2011). Wang et al. (2002) and Li et al. (2008) reported that the NR and GS activity in flag leaves significant decreased after anthesis. Jenner et al. (1991) surmised that the N demands of developing grains induced leaf senescence. In our study, NR showed a general decrease as the leaves aged for most treatments. Also, the NR activity in the N240 treatment was the highest, and it was significantly higher than in the N0 treatment. This is consistent with previous results showing that N starvation causes a significant decrease in the NR activity, but high levels of external nitrate reduce the activity of this enzyme (Lambeck et al., 2012; Balotf et al., 2016). GS activity in flag leaves plays a key role in protein synthesis, especially during the grain filling stage when N is remobilized to the reproductive sinks (Habash et al., 2007). In this study, we found that the GS activity was increased by N addition and showed a general decrease as the leaves aged for all treatments, and the wheat samples with higher GS activities during the grain filling stage also showed increased grain protein content, in agreement with our previous study that used different wheat cultivars (Zhang et al., 2017). One possibility is that N increased GS1 and GS2 expression in flag leaves at early grain filling stage, but the GS expression decreased at mid grain filling stage, especially the GS2 (Zhang et al., 2017).

Dai et al. (2015) reported that the SNA increased sharply with N rate at high N rates in the Loess Plateau of China. In our study, a low level (38.1 kg ha^-1^) of SNA was found in the 0–100 cm soil layer for the N0 treatment, and 68% of the SNA was distributed in the 0–40 cm layer. In the N-fertilized plots, the average SNA increased quadratically from 148.8 to 254.7 kg ha^-1^ in the 0–100 cm soil layer when the N rate was increased from 180 to 300 kg ha^-1^, and these results are similar to those of previous studies. Furthermore, the SNA peak gradually moved down with advancing experimental years, and the SNA gradually decreased during the last two years of the experiment (2015 and 2016). This indicates that the NO_3_-N had moved to the soil layer below 100 cm with irrigation and rainfall, which was due to continuous irrigation for six years and increased precipitation in the last two seasons under the high N application levels. In addition, the increase in the amount of N that accumulated in the plant could be one possible reason behind the higher yields in 2015 and 2016. These results agree with those of Thorburn et al. (2003), who found that N leaching levels were often positively correlated with the amount of irrigation or the N application rate, and there was nitrate leaching in the winter wheat season when each irrigation depth was > 60 mm.

**FIGURE 5:**
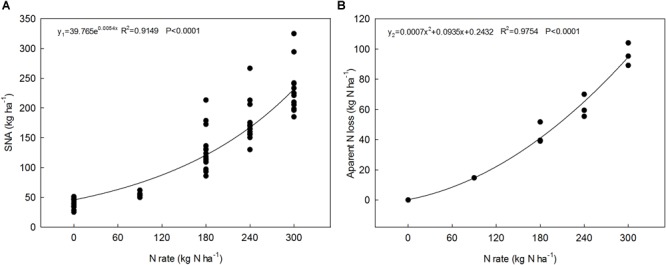
The relationships between SNA in the 0–100 cm soil layer **(A)** and apparent N loss **(B)** with N application rates across all experimental years and planting sites.

One of the methods used to evaluate the rationality of nitrogen management is to estimate the balance between incorporated N, N removed by the crop, and residual inorganic N (Guarda et al., 2004). Our results are consistent with those of Ju et al. (2006) and Sieling and Kage (2006), who found that N surplus is positively correlated with N fertilizer application rate. Our study showed that N rates explain a large part of the variation (∼97%) in N loss for wheat (Figure 5B), which also agrees with the results of Valkama et al. (2013). Soil surpluses and deficits are obviously linked to the N supply and crop N demand. In present study, the N0 and N300 treatments showed a N deficit and surplus, respectively. Several studies have reported that surplus N can be lost by NO_3_-N leaching, which then pollutes the environment (Sieling and Kage, 2006).

On a national or regional scale, PFPN and NAE are important indices of NUE that can be estimated reasonably well, and SNA is negatively related to NUE (Dang et al., 2009). Previous studies have also shown that NAE is significantly reduced at the highest N fertilizer level, and that the average NAE for wheat of 20-25 kg grain increased per kg of N applied worldwide. In our studies, the PFPN and NAE values for wheat varied between 22.9 and 49.9 kg kg^-1^ and between 2.0 and 22.5 kg kg^-1^, respectively, for the different N treatments (Table 2). The significantly lower values of NAE in 2011 (2.2 kg kg^-1^) were due to the higher yield in the N0 treatment at the beginning of the experiment. The lower values of NAE observed in 2013 were due to reduced precipitation during the growing season, which occurred mostly (>70%) at the late grain-filling stage and limited the use of N fertilizer.

The main aim of agricultural production has traditionally been to increase cereal grain yields to feed the expanding population and to meet improved living standards (Liu et al., 2016). Equally importantly, grain protein contents have been increasing in wheat, resulting in improved nutritional quality (Shewry, 2007). In this study, the N rate-yield and N rate-protein content relationships may be valuable models for predicting the N application rates needed to achieve target grain yields, protein contents, and N use efficiencies while reducing N loss and also reducing the environmental costs for sustainable development of agricultural production.

**FIGURE 6:**
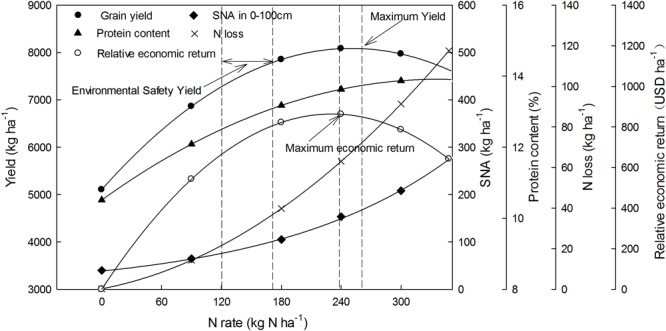
Relationships between grain yield, protein content, apparent N loss, SNA in the 0–100 cm soil layer, and relative economic return with the different N fertilizer application rates.

As shown in the present work, grain yield, grain protein content, SNA in the 0–100 cm soil layer, surplus N, and the relative economic return are all significantly correlated with the N fertilization rate (Figure 6). When the maximum yield (8087 kg ha^-1^) was obtained at 250 kg N ha^-1^, the SNA was as high as 153 kg N ha^-1^ in the 0–100 cm soil layer, corresponding to a PFPN of 32 kg kg^-1^. Clearly, these excessive fertilizer N inputs significantly increase nitrate accumulation in soils, and thus inevitably increase the possibilities of nitrate leaching. The European Union has stipulated that the cumulative NO_3_-N level in the 0–90 cm soil layer should not exceed 90–100 kg N ha^-1^ (Clercq et al., 2001). Therefore, when the SNA in the 0–100 cm soil layer was maintained at environmentally acceptable levels (100 kg N ha^-1^) in this study, the N rate was 171 kg N ha^-1^ and the grain yield was 7787 kg ha^-1^, which was 96.5% of the highest yield. Cui et al. (2005) reported that the apparent N loss for winter wheat was 32 kg ha^-1^ under N-optimized management conditions in the North China Plain. Consistently, the apparent loss was 36.7 kg ha^-1^ in our study at a N rate of 171 kg ha^-1^, and this demonstrates that our models and methods are reliable. Turner et al. (2004) recommend that the grain protein content should be higher than 12.5%, which is the level needed for bread making and for the necessary nutritional quality. Hence, the N application rate should be at least 120 kg ha^-1^ to ensure high grain quality, and that the corresponding yield and SNA reach 7278 and 76 kg ha^-1^, respectively. For common wheat production in North China, the economic benefit is certainly a component need to consider. In this study, the highest relative economic return was obtained at a N rate of 231 kg ha^-1^, which is far higher than the environmentally safe N rate of 171 kg ha^-1^ specified by the European Union. Although this N rate produced higher economic return and yield, N loss and environmental costs also increased because of high N input. It has been well documented that China has been facing larger environmental challenges than many other major countries (Liu and Diamond, 2005), and a great extent of these issues comes from the overuse of N fertilizer in agricultural production (Liu et al., 2013). Therefore, in order to balance grain yield, NUE, N loss, and environmental costs, the recommended N rate should be between 120 and 171 kg N ha^-1^. The ecological environmental benefit is affected by the soil fertility and production conditions. Thus, there is still a need for future research focused on optimizing N management for sustainable agricultural development.

## Conclusion

The grain yield and protein content of winter wheat were found to be significantly related to the N fertilizer application rate in all years and planting sites in this study. The different N application rates significantly affected the chlorophyll content and photosynthetic characteristics of flag leaves at anthesis. Also, the NR and GS activities in flag leaves for the N application treatments were significantly higher than the activities in the N0 treatment after anthesis at three sites. N fertilizer guarantees both wheat yield and quality by regulating these physiological indexes. The SNA in the 0–100cm soil layer and apparent N loss increased sharply with increasing N application rates. An excessive fertilizer N supply triggers nitrate leaching in the intensive crop production system in the North China Plain. In order to balance grain yield, NUE, and N loss, the recommend N application rate is among 120–171 kg N ha^-1^, for these rates, the corresponding yield and apparent N loss were 7278–7787 ka ha^-1^ and 22–37 kg ha^-1^, respectively. The results presented here can help guide winter wheat production, especially in rural areas of China, as well as in similar cropping systems around the world.

## Author Contributions

CW conceived the research and designed the study. GM and WL analyzed the data and wrote the manuscript. GM, PZ, and SL carried out the field measurements and soil analysis. HL, LW, and YX critically reviewed the manuscript. DM and GK assisted with manuscript writing and editing. All authors approved the final version of the manuscript.

## Conflict of Interest Statement

The authors declare that the research was conducted in the absence of any commercial or financial relationships that could be construed as a potential conflict of interest.

## Footnotes

1 http://www.grainoil.com.cn

2 http://www.fert.cn/

## Supplementary Material

FIGURE S1 Annual precipitation by month (October to May) during the wheat growing seasons at the Wenxian **(a)**, Zhengzhou **(b)**, and Kaifeng **(c)** planting sites. The years shown indicate wheat that was harvested that year but was sown the preceding year; for example “2011” represents winter wheat sown in 2010 and harvested in 2011.

TABLE S1 Various physical and chemical properties of the field experiment in 0–20 cm soil in Wenxian, Zhengzhou and Kaifeng.
