# Supplementary material for: Determining the Optimal N Input to Improve Grain Yield and Quality in Winter Wheat With Reduced Apparent N Loss in the North China Plain
Source: Front Plant Sci. 2019 Feb 22;10:181. doi: 10.3389/fpls.2019.00181 (PMC6396033; doi:10.3389/fpls.2019.00181)
Supplement: TABLE S1 — Various physical and chemical properties of the field experiment in 0–20 cm soil in Wenxian, Zhengzhou and Kaifeng. [file Table_1.DOCX]

**TABLE S1** Various physical and chemical properties of the field experiment in 0-20cm soil in Wenxian, Zhengzhou and Kaifeng..

| Site | Bulk density (g cm^-3^) | Organic matter (g kg^-1^) | Total N (g kg^-1^) | Available P (mg kg^-1^) | Available K (mg kg^-1^) | pH |
| --- | --- | --- | --- | --- | --- | --- |
| Wenxian | 1.23 | 18.12 | 1.12 | 22.07 | 157.07 | 8.31 |
| Zhengzhou | 1.28 | 17.47 | 0.84 | 21.09 | 252.56 | 7.91 |
| Kaifeng | 1.24 | 14.85 | 1.17 | 18.83 | 140.28 | 8.01 |
